# Pancreas-Specific Sirt1-Deficiency in Mice Compromises Beta-Cell Function without Development of Hyperglycemia

**DOI:** 10.1371/journal.pone.0128012

**Published:** 2015-06-05

**Authors:** Andreia V. Pinho, Mohammed Bensellam, Elke Wauters, Maxine Rees, Marc Giry-Laterriere, Amanda Mawson, Le Quan Ly, Andrew V. Biankin, Jianmin Wu, D. Ross Laybutt, Ilse Rooman

**Affiliations:** 1 Cancer Division, Garvan Institute of Medical Research, The Kinghorn Cancer Centre, Darlinghurst NSW, Australia; 2 St. Vincent's Clinical School, Faculty of Medicine, UNSW Australia, Randwick NSW, Australia; 3 Diabetes and Metabolism Division, Garvan Institute of Medical Research, Darlinghurst NSW, Australia; 4 Diabetes Research Center, Vrije Universiteit Brussel, Brussels, Belgium; 5 Wolfson Wohl Cancer Research Centre, University of Glasgow, Glasgow, Scotland, United Kingdom; Sanford-Burnham Medical Research Institute, UNITED STATES

## Abstract

**Aims/Hypothesis:**

Sirtuin 1 (Sirt1) has been reported to be a critical positive regulator of glucose-stimulated insulin secretion in pancreatic beta-cells. The effects on islet cells and blood glucose levels when Sirt1 is deleted specifically in the pancreas are still unclear.

**Methods:**

This study examined islet glucose responsiveness, blood glucose levels, pancreatic islet histology and gene expression in Pdx1^Cre^; Sirt1^ex4F/F^ mice that have loss of function and loss of expression of Sirt1 specifically in the pancreas.

**Results:**

We found that in the Pdx1^Cre^; Sirt1^ex4F/F^ mice, the relative insulin positive area and the islet size distribution were unchanged. However, beta-cells were functionally impaired, presenting with lower glucose-stimulated insulin secretion. This defect was not due to a reduced expression of insulin but was associated with a decreased expression of the glucose transporter Slc2a2/Glut2 and of the Glucagon like peptide-1 receptor (Glp1r) as well as a marked down regulation of endoplasmic reticulum (ER) chaperones that participate in the Unfolded Protein Response (UPR) pathway. Counter intuitively, the Sirt1-deficient mice did not develop hyperglycemia. Pancreatic polypeptide (PP) cells were the only other islet cells affected, with reduced numbers in the Sirt1-deficient pancreas.

**Conclusions/Interpretation:**

This study provides new mechanistic insights showing that beta-cell function in Sirt1-deficient pancreas is affected due to altered glucose sensing and deregulation of the UPR pathway. Interestingly, we uncovered a context in which impaired beta-cell function is not accompanied by increased glycemia. This points to a unique compensatory mechanism. Given the reduction in PP, investigation of its role in the control of blood glucose is warranted.

## Introduction

Diabetes affects nearly 350 million people worldwide and of these, 95% of patients have type 2 diabetes, suffering from impaired insulin sensitivity and/or impaired insulin secretion. Pancreatic beta-cells, the most abundant cell type in the islets of Langerhans, are the insulin factories of the body [1].

Sirtuin1 (Sirt1) is a NAD+ dependent protein deacetylase which regulates it’s targets through modifications in the acetylation state of the proteins. There are many different proteins regulated by Sirt1 affecting multiple cellular functions, including glucose metabolism, mitochondrial biogenesis, stress resistance, apoptosis and chromatin silencing [2,3]. Sirt1 is also believed to be a potent protector from ageing-associated pathologies, such as diabetes and cardiovascular disease [3,4].

From previous studies, a consensus has been developed that Sirt1 is highly expressed in beta-cells and is a positive regulator of glucose responsiveness [5,6]. An age-dependent decline in Sirt1 activity has been suggested to contribute to the development of type 2 diabetes [7]. Islets from whole body [5] and pancreas-specific Sirt1-knock out mice [6] show lower insulin secretion in response to glucose, and in agreement, islets from beta-cell specific overexpressing Sirt1-transgenic mice presented improved insulin secretion and glucose tolerance [7,8]. These and other studies are proposing the use of Sirt1 activating drugs, such as the red wine component resveratrol, for healthy ageing and control of diabetes [4]. Nevertheless, there appears to be no consensus on the extent of beta-cell deficiency in the Sirt1 loss context with different experimental models used [5,6,9]. The overall effects of Sirt1 depletion on blood glucose homeostasis have not been accurately assessed, only vaguely interpreted, or even found to be contradictory [5,6,9]. The studies pointed at deregulated expression of Uncoupling protein-2 (Ucp2) [5], and other mitochondrial genes [6] or attributed the effects to a disruption of a Foxa2 transcriptional complex at the Pdx1 promoter [9], essential for beta-cell development and differentiation.

To better understand the role of Sirt1 in blood glucose homeostasis, we comprehensively analyzed pancreatic-specific Sirt1-deficient animals, taking in consideration not only the effects of Sirt1 loss in beta-cell function but also in other endocrine cell types and, in addition, assessed overall effects on blood glucose homeostasis.

## Materials and Methods

### Animals and *in vivo* experimentation

B6.FVB-Tg(Ipf1-cre)6Tuv/J (Pdx1^Cre^), B6;129-Sirt1^tm1Ygu^/J (Sirt1^ex4^) strains were obtained from Jackson Laboratories and bred to create the strain Pdx1^Cre^;Sirt1^ex4F/F^. The reporter strain B6.129X1-Gt(ROSA)26Sor^tm1(EYFP)Cos^/J (R26R^EYFP^) was also acquired from Jackson Laboratories. For all experiments Pdx1^Cre^;Sirt1^+/+^ were used as controls. Blood glucose measurements were performed in fed state or after 16h overnight fasting. Body weight measurements and insulin or glucose tolerance tests were performed after fasting. All animal experiments were approved by the Garvan Animal Ethical Committee (AEC approval #12–52).

### Islet isolation and *ex vivo* insulin secretion assay

Islets were isolated by pancreatic digestion, purified using a Ficoll-paque gradient (GE Healthcare, Chalfont St Giles, UK) and handpicked under a stereomicroscope. Immediately after collection, a fraction of the islets was used for RNA extraction. Remaining islets were washed in Krebs-Ringer HEPES buffer (containing 5 mmol/L NaHCO3, 1 mmol/L CaCl2, 2.8 mmol/L glucose, 10 mmol/L HEPES, and 0.1% BSA). Groups of five islets, with two replicates per animal, were incubated for 1 h at 37°C in 130 μl of Krebs-Ringer HEPES buffer containing 2.8 or 20 mmol/L glucose in a 96 well plate with a V-shaped bottom. Insulin was measured in an aliquot of the buffer by radioimmunoassay in duplicate (Millipore, Billerica, MA).

### Immunofluorescence and morphometric analysis

Mouse whole pancreas tissue was fixed in 4% formalin and processed for paraffin embedding. Antigen retrieval was performed by microwave heating in antigen retrieval solution-citrate buffered (Prosan, Merelbeke, Belgium). Detection was done using fluorochrome-conjugated secondary antibodies (Jackson Laboratory, Westgrove, Pennsylvania, USA) according to manufacturer’s instructions.

The following primary antibodies were used: anti-insulin (Guinea pig Polyclonal—C. Van Schravendijk, Diabetes Research Center, Brussels, Belgium); anti-PP (Rabbit Polyclonal—R.E. Chance, Lilly Research, Indianapolis, USA); anti-Glut2 (Rabbit Polyclonal—Alpha Diagnostic—San Antonio, Texas, USA), anti-KDEL (Mouse monoclonal – Enzo Life Sciences – Farmingdale, NY).

Pictures were acquired with ZEISS LSM7 10 NLO confocal microscope using ZEN 2009 software (Carl Zeiss, Oberkochen, Germany). Quantification of insulin and PP-positive areas and pancreas tissue area (85,29±9,58 mm^2^ spread across 3 non-consecutive sections) was performed with IPlab 4.0 software (Becton Dickinson, San Jose, CA, USA).

### Gene Expression analysis by RT-qPCR

Total RNA was isolated from islets using Purelink RNA Mini Kit (Ambion, Life Technologies, Carlsbad, CA, USA) and cDNA prepared was using Superscript III Reverse transcriptase (Invitrogen, Life Technologies, Carlsbad, CA, USA) according to manufacturer’s instructions. 10ng RNA equivalent was used for qPCR with specific primers (sequences available upon request) in the presence of Power SYBR Master Mix (Invitrogen, Life Technologies, Carlsbad, CA, USA) using the 7900H Fast Real Time PCR System (Life Technologies, Carlsbad, CA, USA). All analyses were done in duplicate. A melting curve analysis was performed for each reaction to control for product quality and specificity. The expression levels were normalized to housekeeping gene expression (Hprt and Cyclophilin A).

### Gene expression arrays and data analysis

Total RNA from whole pancreas of 6-month-old male animals was extracted using Purelink RNA Mini Kit (Ambion, Life Technologies, Carlsbad, CA, USA) and RNA quality was determined using the Agilent 2100 Bioanalyzer (Agilent Technologies, Santa Clara, CA, USA). RNA from three Pdx1^Cre^ and three Pdx1^Cre^;Sirt1^ex4F/F^ pancreata was used for hybridization in GeneChip Mouse Gene 2.0 ST Arrays (Affymetrix, Santa Clara, CA, USA) according with manufacturer’s procedures. R statistical programming language and a number of Bioconductor packages were used for the microarray analysis. Raw data (cell format) were processed using the oligo and pd.mogene.2.0.st packages. Following array quality control, these data were normalized by Robust Multi-array Average (RMA) method and annotated by the mogene20sttranscriptcluster.db package. Probes without gene annotation were excluded from the further analysis. Limma package was applied to identify differentially expressed genes between Pdx1^Cre^ and Pdx1^Cre^;Sirt1^ex4F/F^ groups. Raw data were deposited in the Gene Expression Omnibus database (accession number GSE67239). Only differentially expressed probes, detecting coding regions of the genome, with a p-value <0.01 were included for the pathway analysis. KOBAS 2.0 [10] was applied to perform pathway enrichment analysis. The hypergeometric test was selected to test statistical enrichment of KEGG and Reactome pathways, and only pathways with p-value <0.001 were listed.

### *In vivo* metabolic assays

Intraperitoneal glucose tolerance tests (2 g/kg of glucose solution, Phebra, Lane Cove, NSW, Australia) and intraperitoneal insulin tolerance tests (0.75U/Kg of Actrapid, Novo Nordisk Pharmaceuticals Ltd, Baulkham Hills, NSW, Australia) were performed in conscious mice after 16 h of fasting. Blood samples were taken via tail tipping and blood glucose was measured using an Accu-Chek Performa glucose monitor (Roche Diagnostics, Castle Hill, Australia). Serum insulin levels were analyzed using ELISA (Crystal Chem, Downers Grove, IL).

### Statistics

Results are presented as Mean ± SEM. Data were analyzed by Prism 6.0 using Student *t* test (unpaired *t* test) or two-way ANOVA and Bonferroni post-test and results considered significant when *p*<0.05.

## Results

### Beta-cells from Pdx1^Cre^; Sirt1^ex4F/F^ mice have reduced glucose-stimulated insulin secretion

**Fig 1:**
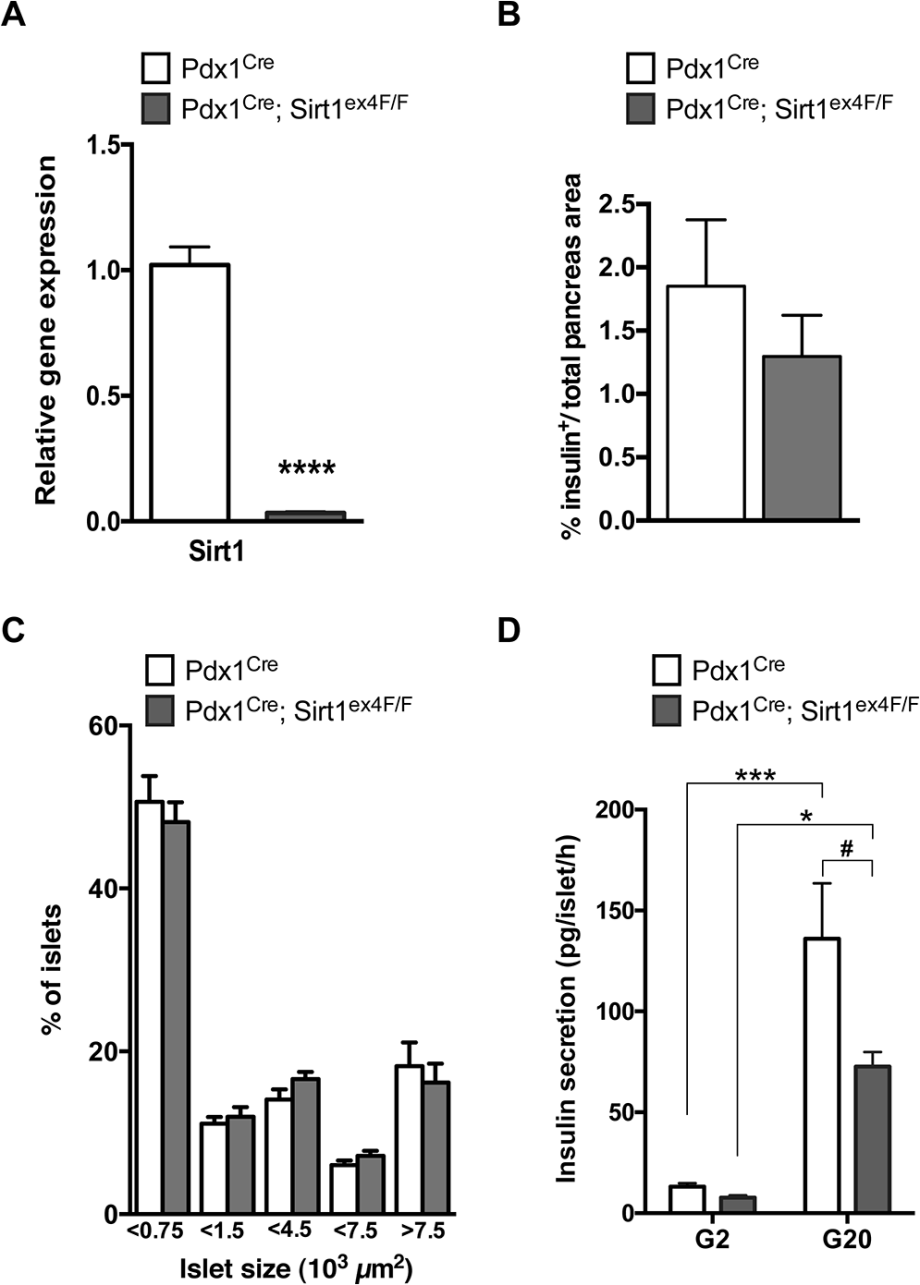
Sirt1-deficient islets present impaired glucose-stimulated insulin secretion but no changes in insulin positive islet area or islet size. A. Sirt1 transcript levels analyzed by RT-qPCR in isolated islets of Pdx1^Cre^ and Pdx1^Cre^; Sirt1^ex4F/F^ mice. Values are relative to the housekeeping gene. (*n*≥6, ****p<0.0001). B. Percentage of islet distribution according to size as measured by morphometry in pancreata from Pdx1^Cre^; Sirt1^ex4F/F^ and control mice. (*n =* 6). C. Relative insulin positive area over total pancreas area in Pdx1^Cre^; Sirt1^ex4F/F^ and control mice as analyzed by morphometry. (*n =* 6). D. *Ex vivo* insulin secretion in islets isolated from 6-month-old Pdx1^Cre^ and Pdx1^Cre^; Sirt1^ex4F/F^ male animals. Batches of five islets were incubated in HEPES-buffered KRB containing 0.1% BSA and 2 mmol/L (G2) or 20 mmol/L glucose (G20) for 1 h. Insulin release was determined by RIA. (*n* = 5–7, *p<0.05, ***p<0.001 for the effect of glucose, # *p*<0.05 for the effect of genotype).

We used a Pdx1^Cre^ driver line to delete Sirt1 exon 4 in the pancreas [11]. Pdx1^Cre^ animals were crossed with the reporter strain R26R^EYFP^ to verify the specificity of Cre-mediated recombination. Cre recombinase activated the expression of the reporter EYFP specifically in pancreatic cells, including exocrine and endocrine tissue (S1 Fig). Six-month-old male Pdx1^Cre^; Sirt1^ex4F/F^ mice expressed a truncated form of *Sirt1* lacking exon 4 which encodes part of the Sirt1 core catalytic domain. Tissue lysates from the total pancreas showed a protein of reduced molecular weight and both total pancreas and isolated endocrine islets presented reduced levels of Sirt1 gene expression (S1 Fig and Fig 1A). Sirt1 gene expression in islets of Sirt1-deficient mice was reduced to 3% of that found in control islets (Fig 1A).

Despite the reduced Sirt1 expression, the relative insulin positive area quantified by immunofluorescence and the distribution of islets according to size was comparable to controls (Fig 1B and 1C). The beta-cell function however was impaired, as evidenced by a reduction in glucose-stimulated insulin secretion (GSIS) at high glucose (20mmol/L glucose, G20) in islets of Pdx1^Cre^; Sirt1^ex4F/F^ animals (72.7 ± 7.2) compared with controls (136.0 ± 27.5) (Fig 1D). The stimulation index itself was not significantly different between control and Sirt1-deficient islets.

These data illustrate that Sirt1-deficient beta-cells secrete less insulin in response to high glucose.

### Beta-cells from Pdx1^Cre^; Sirt1^ex4F/F^ mice express less Glut2, less Glp1r and have a deregulated UPR pathway

**Fig 2:**
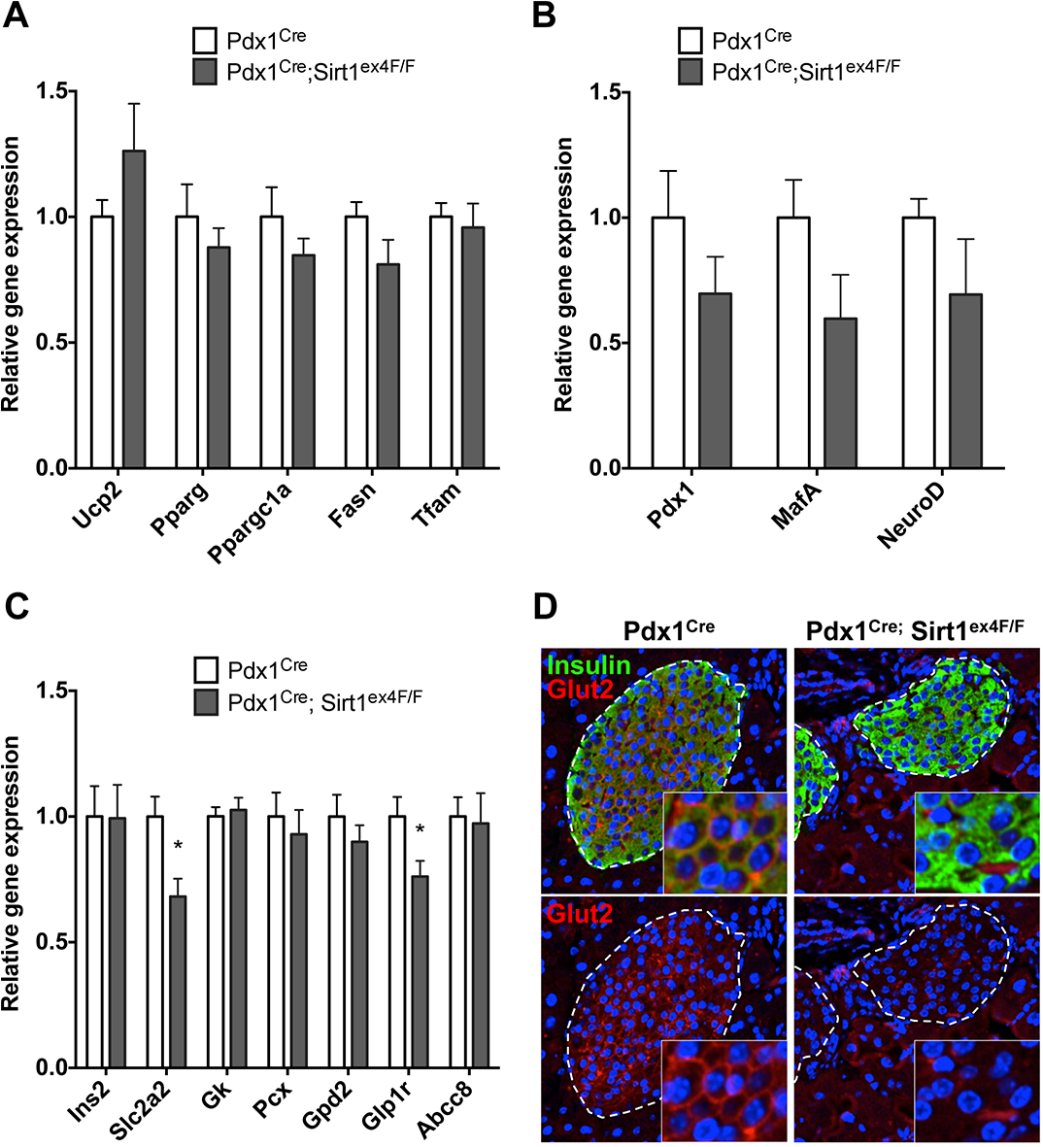
Pdx1^Cre^; Sirt1^ex4F/F^ islets have reduced expression of Slc2a2/Glut2. A. Mitochondrial and fatty acid metabolism gene transcript levels analyzed by RT-qPCR in Pdx1^Cre^ and Pdx1^Cre^; Sirt1^ex4F/F^ isolated islets. Values are relative to the housekeeping gene. (*n*≥6). B. Endocrine transcription factors transcript levels analyzed by RT-qPCR in Pdx1^Cre^ and Pdx1^Cre^; Sirt1^ex4F/F^ isolated islets. Values are relative to the housekeeping gene. (*n*≥6). C. Beta-cell function gene transcript levels analyzed by RT-qPCR in Pdx1^Cre^ and Pdx1^Cre^; Sirt1^ex4F/F^ isolated islets. Values are relative to the housekeeping gene. (*n*≥6, *p<0.05). D. Immunofluorescence staining of Insulin and Slc2a2/Glut2 in islets of Pdx1^Cre^ and Pdx1^Cre^; Sirt1^ex4F/F^ animals. Nuclei are counterstained with DAPI. A representative picture is shown.

We explored the mechanisms underlying the defective GSIS in Sirt1-deficient islets by assessing changes in gene expression. Different to previous reports [5,6], we did not find a significant differential expression in mitochondrial genes or lipid metabolism genes such as *Ucp2*, *Pparg*, *Ppargc1a*, *Fasn* and *Tfam* (Fig 2A). Beta-cell transcription factors showed a tendency for decreased expression (Fig 2B) but, importantly, we did not observe a reduction in the insulin mRNA levels (*Ins2*) or genes implicated in beta-cell function (*Gk*, *Pcx*, *Gdp2*, *Abcc8*) except for the glucose transporter *Slc2a2 (Glut2)* and Glucagon-like peptide 1 receptor (*Glp1r)* (Fig 2C). Immunofluorescence confirmed that Glut2 protein expression is highly reduced at the membrane of Sirt1-deficient beta-cells (Fig 2D and S2 Fig) pointing to a defect in glucose sensing [12]. We noted that the reduction in Glut2 was less pronounced in younger mice (S2 Fig).

**Fig 3:**
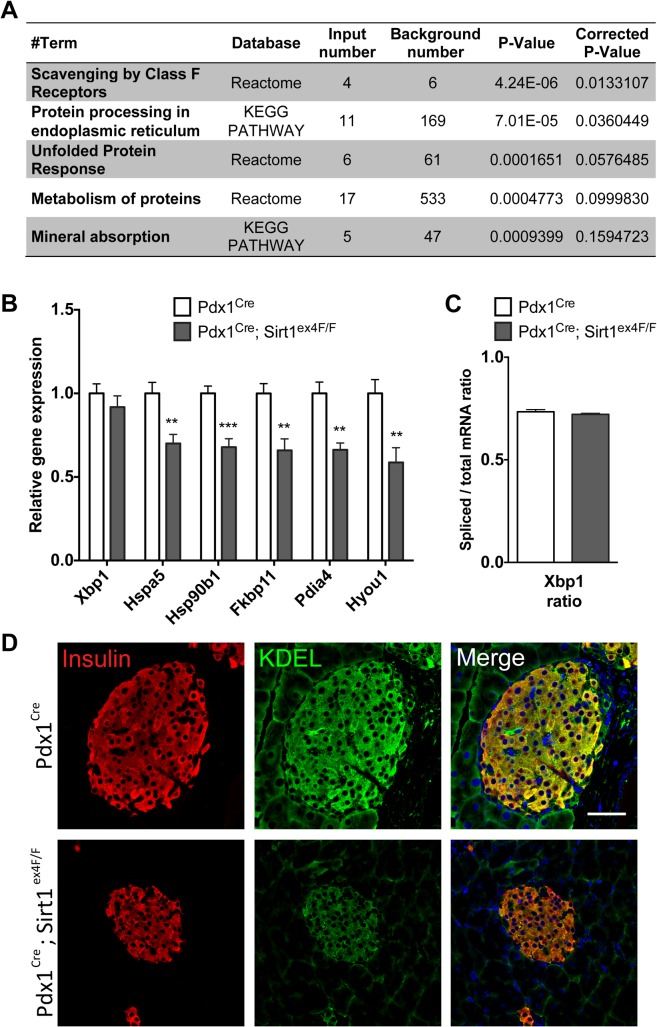
Pdx1^Cre^; Sirt1^ex4F/F^ islets have impaired expression of Endoplasmic Reticulum (ER) chaperone genes involved in the Unfolded Protein Response (UPR) pathway. A. Pathway enrichment analysis of genes differentially expressed in Pdx1^Cre^ vs Pdx1^Cre^;Sirt1^ex4F/F^ whole pancreata. (*n* = 3, p<0.001). B. UPR pathway gene transcript levels analyzed by RT-qPCR in Pdx1^Cre^ and Pdx1^Cre^; Sirt1^ex4F/F^ isolated islets. Values are relative to the housekeeping gene. (*n*≥6, **p<0.01). C. *Xbp1* Splicing. *Xbp1* cDNA was amplified by PCR and digested with *PstI*. Spliced *Xbp1* lacks the restriction site and remains intact. Spliced (intact) and unspliced (cut) *Xbp1* were quantified by densitometry. The value obtained for spliced *Xbp1* was expressed as a ratio of the total (spliced + unspliced) *Xbp1* mRNA level for each sample. (*n*≥6). D. Immunofluorescence staining of Insulin and Bip/Hspa5 and Grp94/Hsp90b1 (anti-KDEL antibody) in islets of Pdx1^Cre^ and Pdx1^Cre^; Sirt1^ex4F/F^ animals. Nuclei are counterstained with DAPI. A representative picture is shown.

To uncover other Sirt1-regulated mechanisms, we performed a gene expression microarray analysis comparing pancreata from the 6-month-old Sirt1–deficient mice and their controls (n = 3 per group). Pathway enrichment analysis of the 257 genes differentially expressed between the two groups (p<0.01) revealed that ‘Protein processing in endoplasmic reticulum’ was one of the two pathways significantly affected in Sirt1-deficient animals (Corrected P-value<0.05) (Fig 3A). Along the same line, ‘Unfolded Protein Response’ (UPR) showed a trend for enrichment (Fig 3A). These are mechanisms well known to regulate beta-cell function [13] that we further explored. Indeed, in the islets of Pdx1^Cre^; Sirt1^ex4F/F^ mice we found reduced gene expression of several endoplasmic reticulum (ER) chaperones, including *Hspa5*, *Hsp90b1*, *Pdia4* and *Hyou1*, and the protein folding gene *Fkbp11* (Fig 3B). Protein expression analysis by immunofluorescence using an antibody anti-KDEL, a common motif in Bip/Hspa5 and Grp94/Hsp90b1 protein, showed reduced levels of these proteins in Sirt1-deficient beta-cells (Fig 3D). Again, the reduction in expression became more pronounced with age (S3 Fig). Expression levels of the spliced form of X-box binding protein 1 (Xbp-1) that can drive the transcription of ER chaperones [14] were unchanged (Fig 3C).

Thus, our analyses uncovered that the defective GSIS of Sirt1-deficient beta-cells may be attributed to impaired glucose sensing together with deregulation of the UPR pathway.

### Pdx1^Cre^; Sirt1^ex4F/F^ mice do not develop hyperglycemia

**Fig 4:**
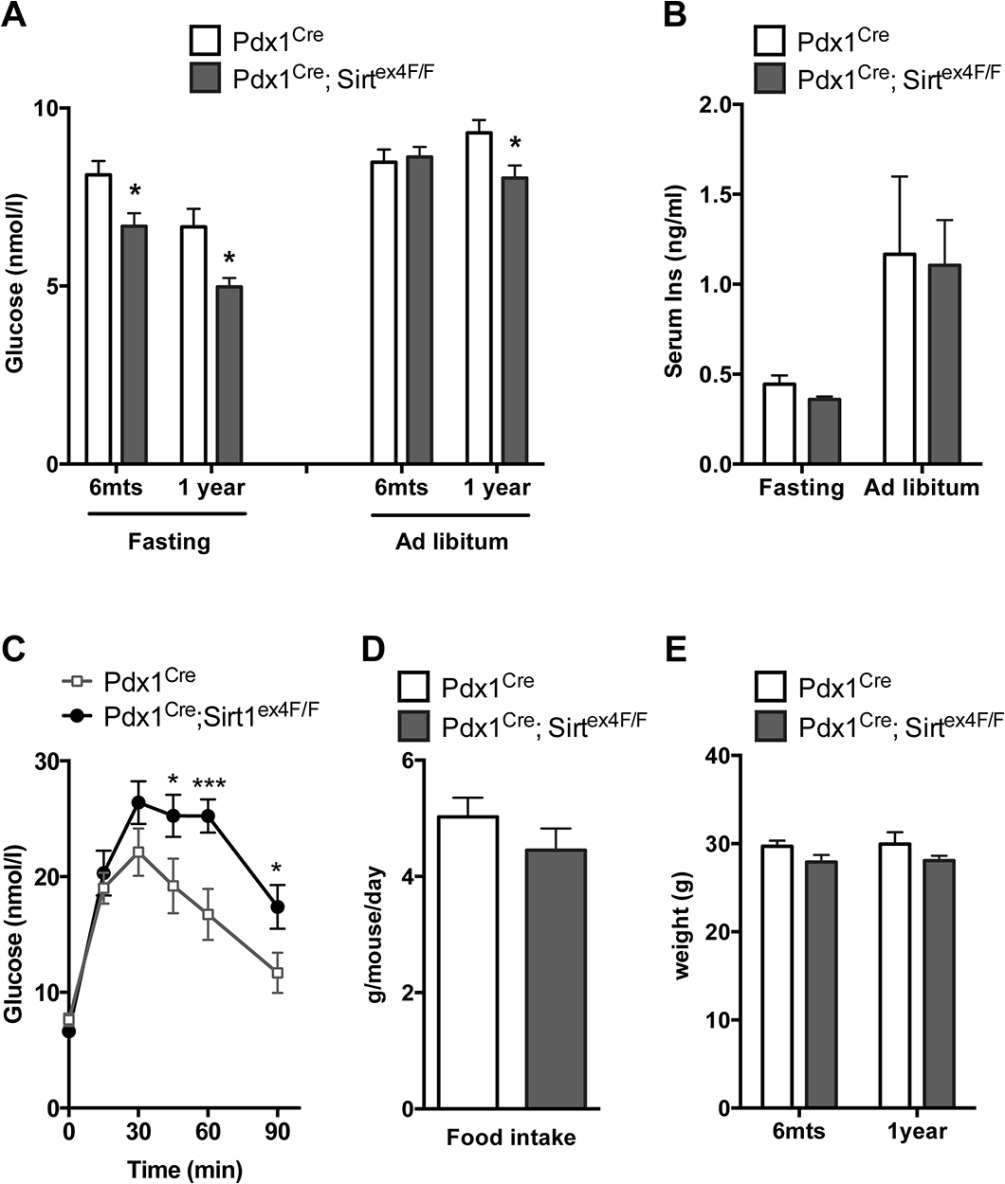
Pdx1^Cre^; Sirt1^ex4F/F^ animals have impaired insulin secretion but are not hyperglycaemic. A. Blood glucose levels of 6-month (*n≥*9) and 1-year-old (*n≥*5) Pdx1^Cre^ and Pdx1^Cre^; Sirt1^ex4F/F^ male animals after 16h of fasting or when fed *ad libitum* (*p<0.05). B. Serum insulin levels in 6-month-old Pdx1^Cre^ and Pdx1^Cre^; Sirt1^ex4F/F^ male animals after 16h of fasting or when fed *ad libitum* measured by ELISA (*n≥*9). C. *In vivo* glucose tolerance test in Pdx1^Cre^ and Pdx1^Cre^; Sirt1^ex4F/F^ male animals. After 16h of fasting, animals were given an intraperitoneal injection of 2 g/kg of glucose solution and blood glucose was measured at different time points. (*n* = 6, *p<0.05, ***p<0.001). D. Quantification of daily food intake in Pdx1^Cre^ and Pdx1^Cre^; Sirt1^ex4F/F^ male animals. (n = 10). E. Body weights of 6-month (n≥14) and 1-year-old (n≥5) Pdx1^Cre^ and Pdx1^Cre^; Sirt1^ex4F/F^ male animals after 16h of fasting. (*p<0.05).

Reduction of Glut2 expression and induction of ER stress often are attributed to glucose toxicity [13,15]. However, the Pdx1^Cre^; Sirt1^ex4F/F^ mice do not have higher, but lower fasting glycemia when assessed at different ages (Fig 4A). These results were intriguing given the impaired GSIS. Even when fed *ad libitum*, their glycemia was not higher than that of the controls at 6 months of age and was significantly lower at 1 year of age (Fig 4A). Serum insulin levels were similar between Pdx1^Cre^; Sirt1^ex4F/F^ and controls both in fasting and *ad libitum* conditions (Fig 4B). Only when challenged by an intraperitoneal (i.p.) glucose bolus, we found decreased glucose tolerance in the mice (Fig 4C). No difference in glycemia was measured when insulin was administered i.p. (S4 Fig). These results indicate that the Sirt1-deficient mice have impaired beta-cell function, which becomes apparent under supra-physiological challenges and not when the mice have normal food intake. Of note, the daily food consumption in the *ad libitum* group did not differ between controls and Sirt1-deficient mice (Fig 4D) and, accordingly, body weight did not differ (Fig 4E). All mice were in healthy condition during the period of the study (up to 1 year).

### Pdx1^Cre^; Sirt1^ex4F/F^ mice present a reduced number of islet PP-cells

**Fig 5:**
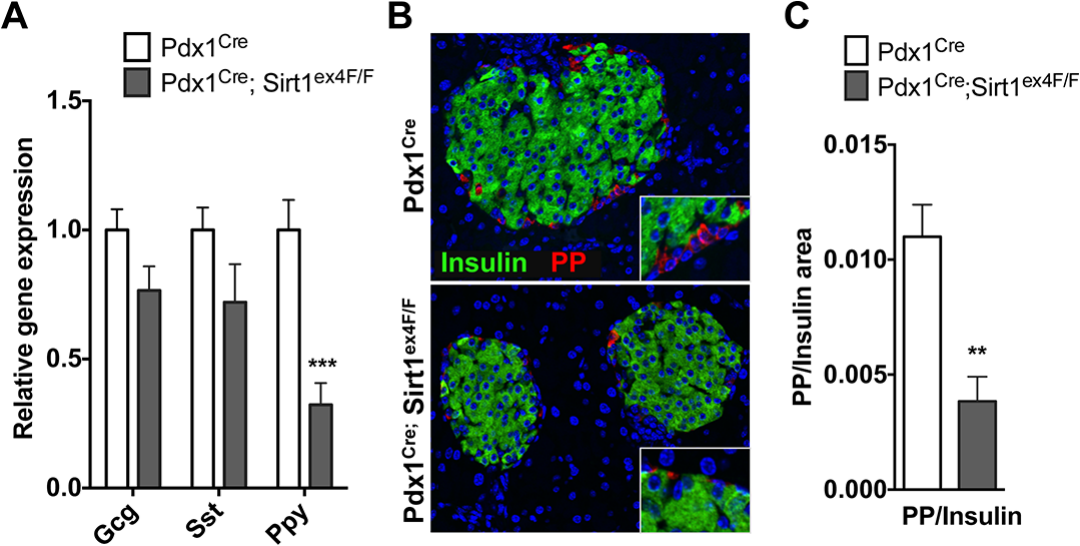
Sirt1 deficient islets present reduced expression of pancreatic polypeptide and a marked reduction of PP-cell number. A. Endocrine hormone transcript levels analyzed by RT-qPCR in Pdx1^Cre^ and Pdx1^Cre^; Sirt1^ex4F/F^ isolated islets. Values are relative to the housekeeping gene. (*n*≥6, **p<0.01, ***p<0.001). B. Immunofluorescence staining of Insulin and Pancreatic Polypeptide (PP) in islets of Pdx1^Cre^ and Pdx1^Cre^; Sirt1^ex4F/F^ animals. Nuclei are counterstained with DAPI. A representative picture is shown. C. Quantification of PP^+^ area relative to Insulin^+^ area performed with IPlab 4.0 software. (*n* = 6, **p<0.01).

To further explore the context of the observations above on blood glucose homeostasis, we assessed the gene expression of other islet hormones (Fig 5A). Whereas there are tendencies for decreased expression in glucagon (*Gcg*) and somatostatin (*Sst*), only polypeptide Y (*Ppy*) gene expression was significantly reduced. In agreement, the area of pancreatic polypeptide (PP) positive cells relative to insulin area was strongly reduced in Pdx1^Cre^; Sirt1^ex4F/F^ animals (0.0038 ± 0.0011) compared with controls (0.011 ± 0.0014) (Fig 5B and 5C). The latter result underscores that pancreatic Sirt1-deficiency not only impacts on beta-cell function but also on the number of PP-cells.

## Discussion

In this study we showed that Pdx1^Cre^; Sirt1^ex4F/F^ mice presented with a normal insulin producing cell mass and normal islet size but impaired islet function. Our data confirm two previous reports from Bordone *et al*. and Luu *et al*. [5,6] where a similar phenotype was described and, in addition, no impairment of ion channel function or cellular depolarization, or no effect on total insulin content was found. In contrast to these two studies and to our current findings, Wang *et al*. reported tremendous effects of Sirt1 depletion in the pancreas, with striking reduction in islet number, and consequently high mortality due to severe hyperglycemia [9]. We note that the latter study used a deletion of exons 5 and 6 of the Sirt1 gene. Bordone *et al*. also used deletion of exons 5/6 [5], whereas Luu *et al*. [6] and ourselves used a deletion of Sirt1 exon 4. Exons 4–8 code for the core catalytic domain of Sirt1 but the exon 4 deletion results in a truncated protein that is expressed at low levels (S1B and S1C Fig) whereas in the exons 5/6 deletion studies there is no expression of a truncated protein [16,17]. It was already noticed in whole body knock-out and liver-specific knock-out studies that the exons 5/6 deletion has more severe consequences than the exon 4 deletion [16,18]. Wang *et al*. also used a different Pdx1 line to drive Cre recombinase expression to the pancreas, i.e. a Cre-driver from a 5.5kb fragment containing the Pdx1 promoter that is expressed in all epithelial cells in the pancreas [16]. We used a Cre-driver based on a 4.5 kb fragment of the same promoter [19] that results in a more mosaic expression, especially in the exocrine tissue (S1 Fig). The vast majority of the endocrine islet cells, though, seem to have recombination, in agreement with the near complete suppression of Sirt1 expression (Fig 1A). Still, the differences in experimental design could have contributed to the observed discrepancies. Nonetheless, given the hyperglycemic state of the mice in the Wang *et al* study, their reported changes in insulin cell differentiation may be a consequence rather than a cause of diabetes since it is well known that high glucose negatively affects beta-cell differentiation [15,20,21].

Whereas our observation on the GSIS defect agreed with Bordone *et al*. [5] who used the whole body Sirt1^exon5/6^ knock out mouse, they attributed this effect to altered Ucp2 expression, which we could not confirm, and neither did Luu *et al*. who studied loss of Sirt1 in a beta-cell line MIN6 [6]. We did not observe a significant change in the expression of mitochondrial and lipid metabolism genes such as *Tfam*, *Pparg*, *Ppargc1a* and *Fasn* in contrast to the findings in MIN6 cells[6]. Of note, our study uses a pancreas-specific Sirt1 deficiency and isolated islets thereof, which provides a more physiological model than the insulinoma-derived MIN6 cell line and which eliminates effects from Sirt1 deletion outside the pancreas.

We discovered that the expression of the glucose transporter Slc2a2/Glut2, essential for adequate response to glucose [12], was strongly reduced in the beta-cells of Pdx1^Cre^;Sirt1^ex4F/F^ mice. Moreover, UPR signaling that protects the cells against misfolded proteins in the ER [13], was attenuated as evidenced by reduced expression of ER chaperones. In the liver, Sirt1 was already found to be a negative regulator of UPR signaling and overexpression of Sirt1 alleviated ER stress [22]. Beta-cells depend on efficient ER function as they synthesize and secrete large amounts of insulin. Indeed, the loss of adaptive UPR has been associated with altered beta-cell function, loss of differentiation and progression to diabetes [13]. The expression of spliced Xbp1, which is an important regulator for a subset of UPR target genes [14], was not altered in Sirt1-deficient animals. Mice with loss of this key regulator of the UPR have also been shown to present a reduction of Slc2a2/Glut2 in the membrane of beta-cells [23], similar to our observations. It could still be that Xbp1 acetylation was affected in the Sirt1-deficient cells since Sirt1 regulates XBP1 through deacetylation [24], a hypothesis which could be pursued in future studies. In conclusion, we provided insights implying Sirt1 in the regulation of glucose sensing and UPR signaling in islets, mechanisms that were previously unrecognized. Studying the underlying effects of altered acetylation and their exact inter-regulatory mechanisms will be important in the future.

Interestingly, we clearly showed that Pdx1^Cre^; Sirt1^ex4F/F^ mice do not develop hyperglycemia when kept under normal conditions and even present lower glycemia when fasting and in 1 year old *ad libitum* conditions. This eliminates hyperglycemia as a causal factor in the reduction of Glut2 expression and in the induction of ER stress. In the full body Sirt1 knock-out mouse, the absence of elevated blood glucose was noted and attributed to possible effects of Sirt1 deficiency in peripheral tissues [5], a hypothesis that we can refute in our model since we studied a pancreas-specific deletion of Sirt1. In a recent report, where Sirt1 deletion in the adult islets was obtained by use of a tamoxifen-inducible Pdx1^CreER^ driver, a trend of lower blood glucose levels is shown but without further discussion [6]. Again, this observation corroborates our finding in a slightly different experimental model, i.e. Pdx1^CreER^ that specifically recombines in the pancreatic islets upon tamoxifen administration versus our Pdx1^Cre^ where the pancreatic epithelium is targeted from embryonic development on. It was only Wang *et al*. whoreported severe hyperglycemia upon Sirt1 deletion [9]. In a gain of function approach with beta-cell-specific Sirt1-overexpressing mice, insulin secretion is increased and the mice show improved glucose tolerance but, again, no difference was observed in unchallenged blood glucose levels [8]. In conclusion, the present study is the first to clearly highlight that pancreas-specific Sirt1 loss does not result in hyperglycemia, despite having compromised beta-cell function. This suggests that there may be alterations in the other (non-beta) endocrine cells that offer a counter regulation.

Of all the islet hormones, PP was remarkably decreased in the Pdx1^Cre^; Sirt1^ex4F/F^. A novel role for PP-cells may emerge, a cell type of which not much is documented besides that these cells release PP in response to hypoglycemia. The PP hormone mainly regulates gastric juice secretion and reduces appetite. However, with the lower PP in the Sirt1-deficient mice, we did not measure a difference in food intake and, accordingly, no difference in body weights. Interestingly, at young age, mice deficient in Npy6r, the main PP receptor in the brain, present with improved glucose homeostasis despite lower serum insulin concentrations [25]. We speculate that lower PP secretion in the Sirt1-deficient mice could contribute to the control of blood glucose levels.

This study revealed a collection of novel mechanistic insights on the complex role of Sirt1 in pancreatic islets, which is important to advance therapeutic approaches in diabetes.

## Supporting Information

S1 Fig A. Lineage tracing of EYFP expression using immunhohistochemistry (anti-GFP antibody, rabbit polyclonal, A11122, Molecular probes) in pancreatic tissue from Pdx1^Cre^ and Pdx1^Cre^; R26R^EYFP^ mice, shows that Cre recombinase is active specifically in pancreatic cells (endocrine and exocrine). B. Sirt1 transcript levels analysed by RT-qPCR in total pancreas of Pdx1^Cre^ and Pdx1^Cre^; Sirt1^ex4F/F^ animals. Values are relative to the housekeeping gene. (n = 4, **p<0.01). C. Protein expression analysed by western blot in total pancreas of 6-month-old Pdx1^Cre^ and Pdx1^Cre^; Sirt1^ex4F/F^ mice. Anti-Sirt1 (rabbit polyclonal, HPA006295, Sigma-Aldrich) and anti-Beta-actin (mouse monoclonal, AC5441, Sigma-Aldrich) antibodies were used.
(TIF)

S2 Fig Immunofluorescence staining of Insulin and Slc2a2/Glut2 in islets of Pdx1^Cre^;Sirt1^ex4F/F^ animals compared with control animals at different ages. Nuclei are counterstained with DAPI. A representative picture is shown.
(TIF)

S3 Fig Immunofluorescence staining of Insulin and Bip/Hspa5 and Grp94/Hsp90b1 (anti-KDEL antibody) in islets of Pdx1^Cre^; Sirt1^ex4F/F^ animals compared with control animals at different ages. Nuclei are counterstained with DAPI. A representative picture is shown.
(TIF)

S4 Fig *In vivo* insulin tolerance test in Pdx1^Cre^ and Pdx1^Cre^; Sirt1^ex4F/F^ male animals. After 16h of fasting, animals were given an intraperitoneal injection of 0.75U/kg of insulin and blood glucose was measured at different time points. (*n* = 6).
(TIF)
