# Supplementary figures and images for: Pancreas-Specific Sirt1-Deficiency in Mice Compromises Beta-Cell Function without Development of Hyperglycemia
Source: PLoS One. 2015 Jun 5;10(6):e0128012. doi: 10.1371/journal.pone.0128012 (PMC4457418; doi:10.1371/journal.pone.0128012)

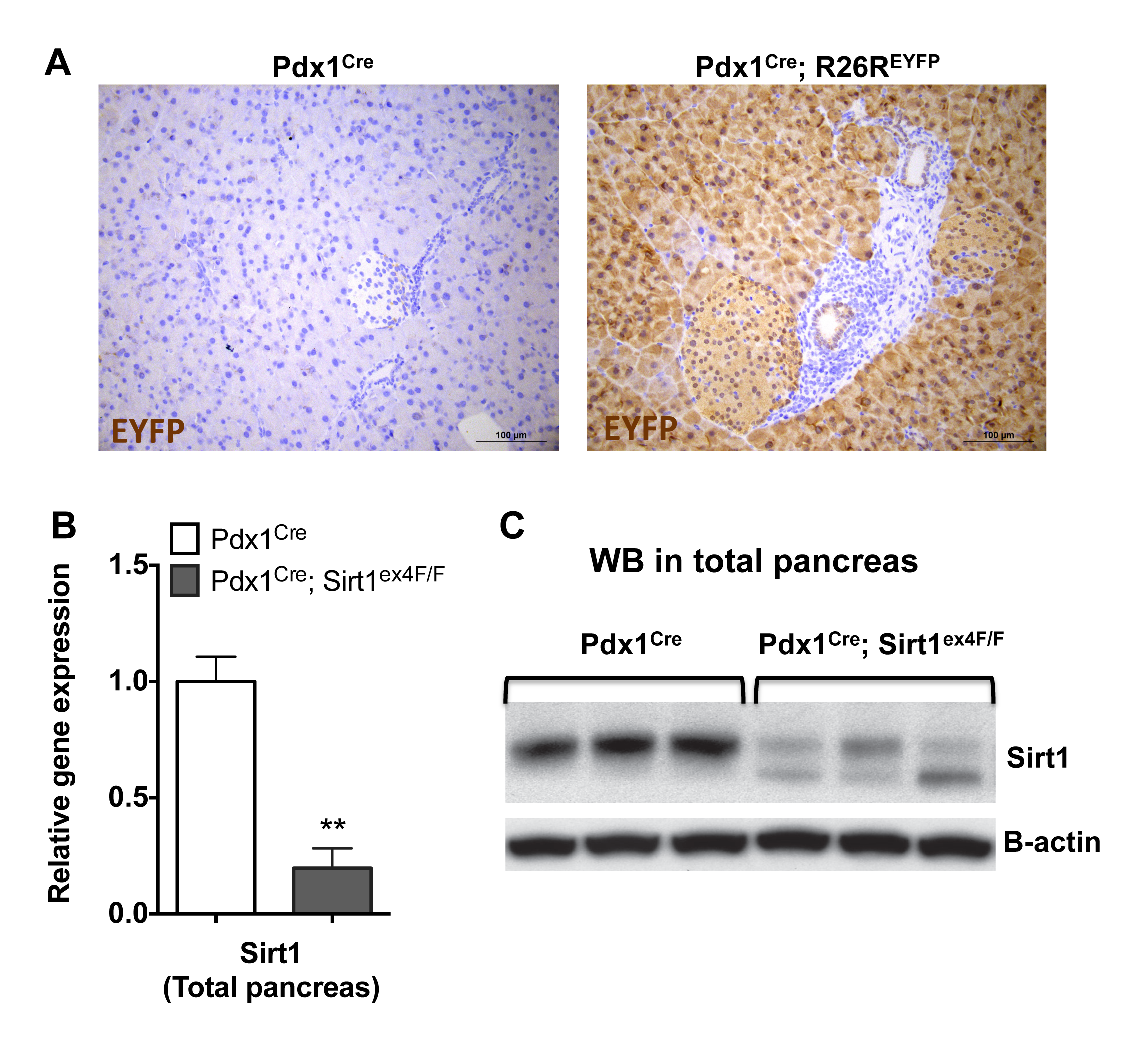

Supplement: S1 Fig — A. Lineage tracing of EYFP expression using immunhohistochemistry (anti-GFP antibody, rabbit polyclonal, A11122, Molecular probes) in pancreatic tissue from Pdx1Cre and Pdx1Cre; R26REYFP mice, shows that Cre recombinase is active specifically in pancreatic cells (endocrine and exocrine). B. Sirt1 transcript levels analysed by RT-qPCR in total pancreas of Pdx1Cre and Pdx1Cre; Sirt1ex4F/F animals. Values are relative to the housekeeping gene. (n = 4, **p<0.01). C. Protein expression analysed by western blot in total pancreas of 6-month-old Pdx1Cre and Pdx1Cre; Sirt1ex4F/F mice. Anti-Sirt1 (rabbit polyclonal, HPA006295, Sigma-Aldrich) and anti-Beta-actin (mouse monoclonal, AC5441, Sigma-Aldrich) antibodies were used. (TIF) [file pone.0128012.s001.tif]

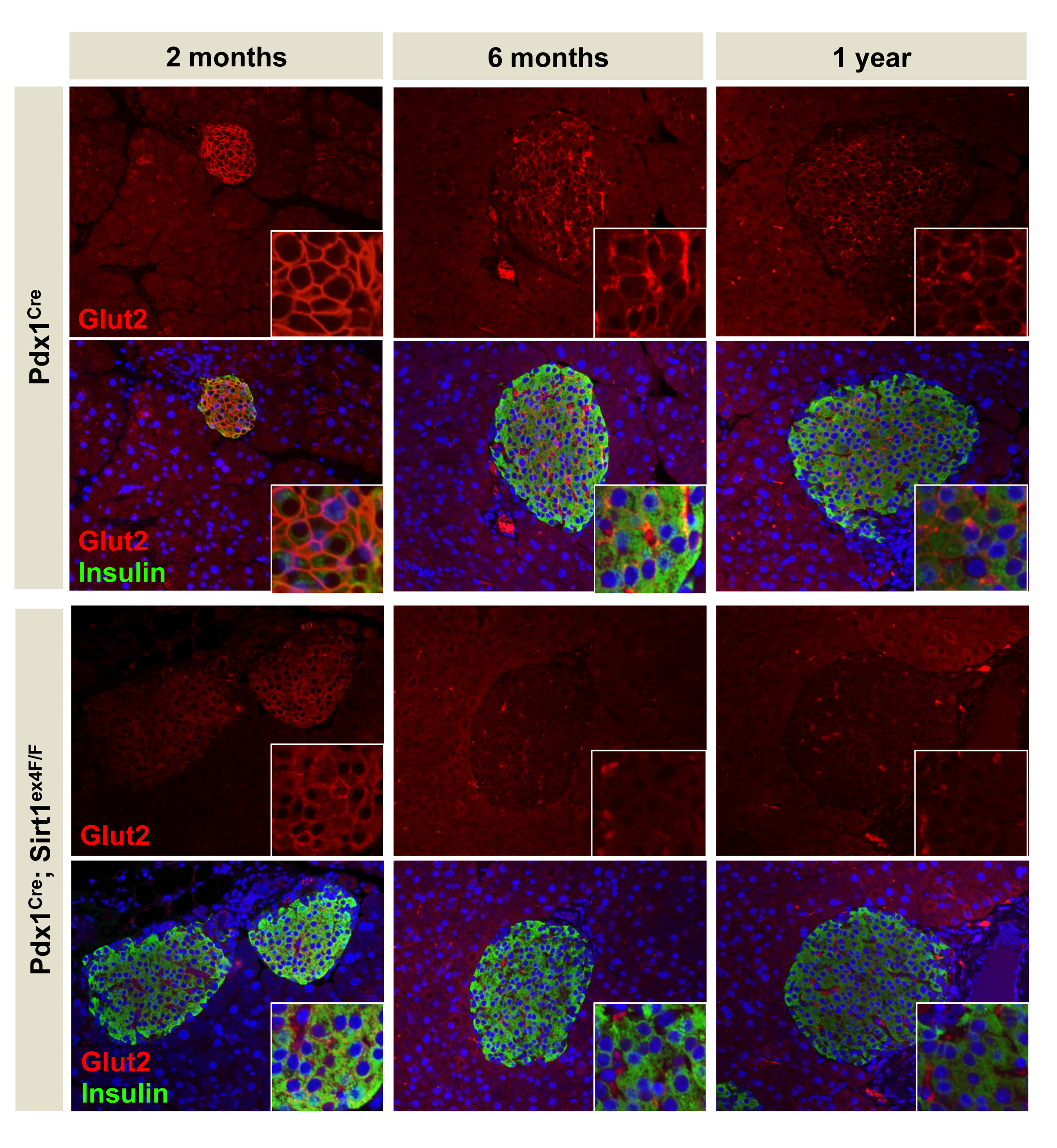

Supplement: S2 Fig — Nuclei are counterstained with DAPI. A representative picture is shown. (TIF) [file pone.0128012.s002.tif]

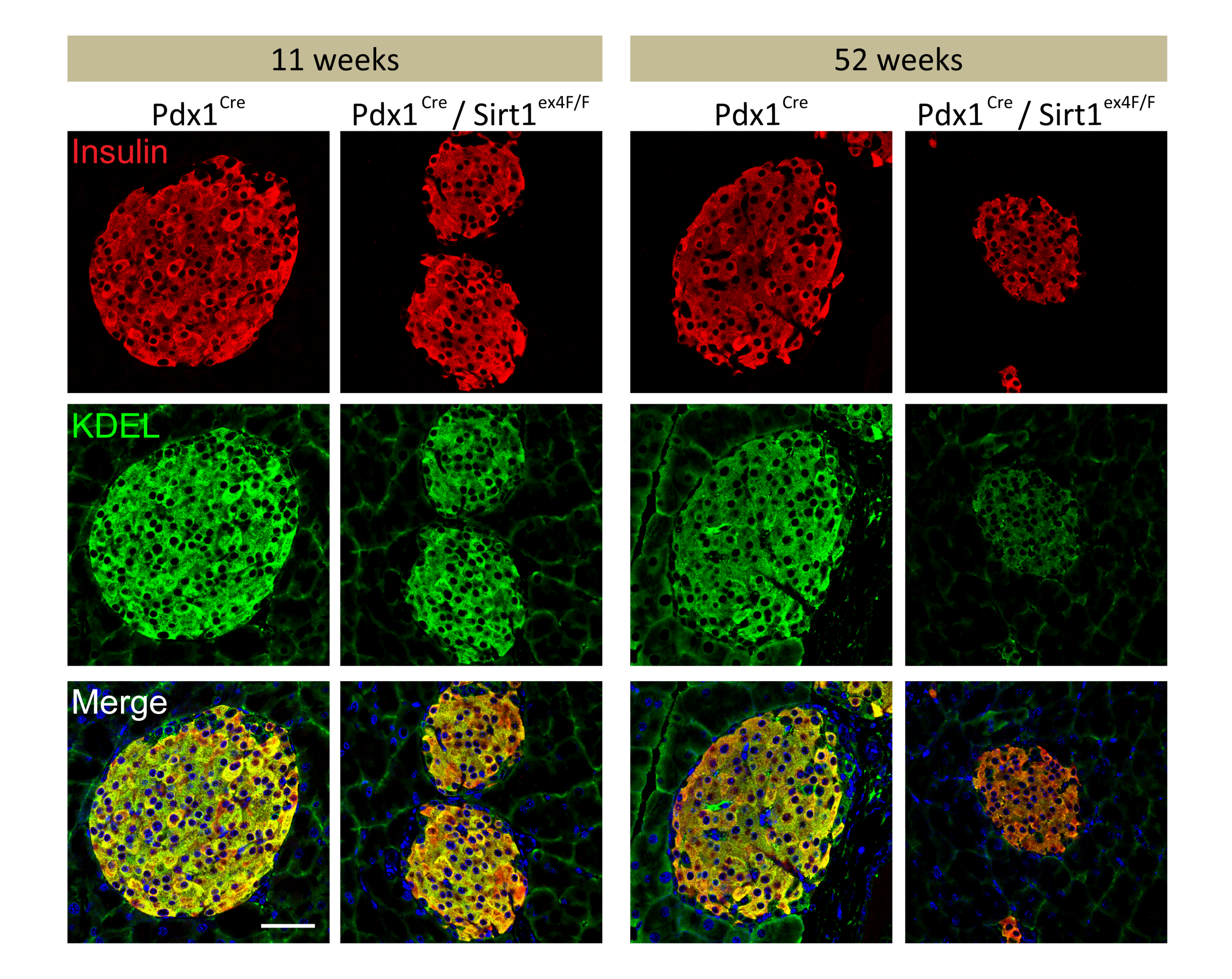

Supplement: S3 Fig — Nuclei are counterstained with DAPI. A representative picture is shown. (TIF) [file pone.0128012.s003.tif]

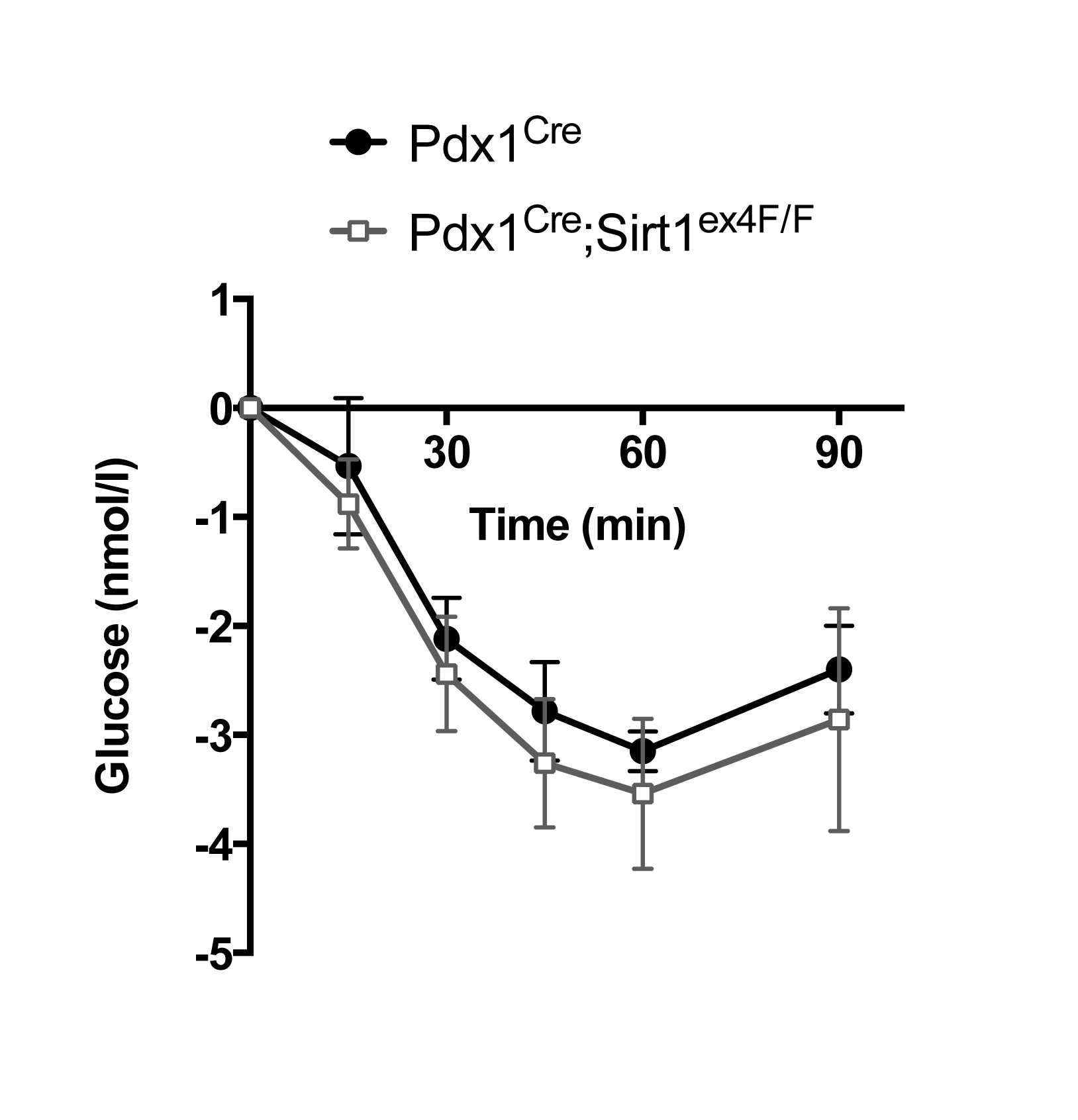

Supplement: S4 Fig — After 16h of fasting, animals were given an intraperitoneal injection of 0.75U/kg of insulin and blood glucose was measured at different time points. (n = 6). (TIF) [file pone.0128012.s004.tif]
